# Renal involvement in eosinophilic granulomatosis with polyangiitis

**DOI:** 10.3389/fmed.2023.1244651

**Published:** 2023-09-18

**Authors:** Francesco Reggiani, Vincenzo L’Imperio, Marta Calatroni, Fabio Pagni, Renato Alberto Sinico

**Affiliations:** ^1^Department of Biomedical Sciences, Humanitas University, Pieve Emanuele, Italy; ^2^Nephrology and Dialysis Unit, IRCCS Humanitas Research Hospital, Rozzano, Italy; ^3^Department of Medicine and Surgery, Pathology, University of Milano-Bicocca, IRCCS (Scientific Institute for Research, Hospitalization and Healthcare) Fondazione San Gerardo dei Tintori, Monza, Italy

**Keywords:** EGPA, rapidly progressive glomerulonephritis, ANCA antibodies, immunosuppressive therapies, glucocorticoids, necrotizing vasculitis, interstitial infiltration

## Abstract

Eosinophilic granulomatosis with polyangiitis (EGPA) is a necrotizing vasculitis, which typically affects small-to medium-sized blood vessels. It is characterized by the presence of tissue infiltrates rich in eosinophils, along with the formation of granulomatous lesions. About 40% of cases have positive anti-neutrophil cytoplasm antibodies (ANCA), with predominant perinuclear staining, and anti-myeloperoxidase (anti-MPO) specificity in about 65% of cases. Typical manifestations of EGPA include the late onset of asthma, nasal and sinus-related symptoms, peripheral neuropathy, and significant eosinophilia observed in the peripheral blood. In contrast to granulomatosis with polyangiitis and microscopic polyangiitis, renal involvement in EGPA is less frequent (about 25%) and poorly studied. Necrotizing pauci-immune crescentic glomerulonephritis is the most common renal presentation in patients with ANCA-positive EGPA. Although rarely, other forms of renal involvement may also be observed, such as eosinophilic interstitial nephritis, mesangial glomerulonephritis, membranous nephropathy, or focal sclerosis. A standardized treatment for EGPA with renal involvement has not been defined, however the survival and the renal outcomes are usually better than in the other ANCA-associated vasculitides. Nonetheless, kidney disease is an adverse prognostic factor for EGPA patients. Larger studies are required to better describe the renal involvement, in particular for patterns different from crescentic glomerulonephritis, and to favor the development of a consensual therapeutic approach. In this article, in addition to personal data, we will review recent findings on patient clinical phenotypes based on ANCA, genetics and the impact of biological drugs on disease management.

## 1. Introduction

Eosinophilic granulomatosis with polyangiitis (EGPA), previously referred to as Churg-Strauss syndrome, is a condition associated with asthma and eosinophilia and characterized by eosinophil-rich and necrotizing granulomatous inflammation, frequently affecting the respiratory tract, and necrotizing vasculitis predominantly affecting small to medium vessels (1–4). This rare autoimmune disease has an incidence ranging from 0.5 to 4.2 cases per million person-years, and a global prevalence estimated to be between 10 and 18 cases per million inhabitants (1, 5). The mean age at diagnosis is 50 years and there are no differences in incidence between men and women (6).

EGPA is classified as a antineutrophil cytoplasmic antibody (ANCA)-associated vasculitis (AAV) even though ANCA are present in a minority of cases (2). Indeed, its clinical and biochemical presentation is different from granulomatosis with polyangiitis (GPA) and microscopic polyangiitis (MPA). EGPA is characterized by three phases: 1) a prodromal “allergic” phase with asthma, allergic rhinitis and sinusitis, 2) a subsequent variable period of up to 8–10 years with eosinophilia and pulmonary infiltrates, eosinophilic cardiomyopathy and gastrointestinal involvement, and 3) a third phase in which the features of vasculitis, as palpable purpura and glomerulonephritis, occur in association with ANCA positivity, usually with anti-myeloperoxidase (anti-MPO) specificity (1, 5, 7). However, the phases may not follow this order, there may be an overlap between different phases and some patients may not exhibit vasculitic complications (8).

The clinical presentation of EGPA is heterogeneous and current evidence suggests that two different phenotypes are associated with ANCA positivity or negativity. Approximately 40% of individuals with EGPA are found to be positive for ANCA, with predominant perinuclear staining and anti-MPO specificity in about 65% of cases (6, 7, 9). The prodromal phase of EGPA is commonly characterized by asthma and ear-nose-throat (ENT) disease. Asthma is observed in over 90% of patients, while ENT disease occurs in approximately 60–80% of individuals. Importantly, both asthma and ENT disease are prevalent in both ANCA-positive and ANCA-negative patients (6). However, the classical manifestations of vasculitis, such as glomerulonephritis, peripheral neuropathy, and purpura, tend to be more common in ANCA-positive patients, while cardiac involvement and gastroenteritis, addressed as eosinophilic features, are more frequent in ANCA-negative patients (6, 7, 9, 10).

Due to the heterogeneity of the clinical presentation, the diagnosis of EGPA can be challenging. None of the disease features alone can be considered pathognomonic. Moreover, EGPA commonly presents as a phasic disease, where both clinical and pathological findings vary based on the specific anatomical site affected and the phase of the illness. Churg and Strauss originally described allergic granulomatosis and angiitis in 1951 based on a study of 13 patients, of which 11 were autopsied. These patients exhibited severe asthma and shared common histological features, including blood and tissue eosinophilia, necrotizing vasculitis, and necrotizing granulomas centered around necrotic eosinophils. However, it is important to note that not all patients displayed all three of these pathological criteria, making them unreliable for clinical diagnosis in the majority of cases (11).

The diagnosis of EGPA should be considered in individuals who have a history of asthma, chronic rhinosinusitis, and eosinophilia, and subsequently develop end-organ involvement, particularly peripheral neuropathy, lung infiltrates, and cardiomyopathy, but also skin, gastrointestinal and kidney involvement (4, 12). Although many attempts to develop diagnostic criteria have been made (13–15), none of them has been validated for diagnosis (12). In the MIRRA trial eligibility criteria to define EGPA have been proposed, but they still need validation. These criteria encompassed asthma, eosinophilia, and the occurrence of at least two of the following manifestations: histological confirmation of eosinophilic vasculitis, perivascular eosinophilic infiltration, or eosinophil-rich granulomatous inflammation; neuropathy; pulmonary infiltrates; sino-nasal abnormalities; cardiomyopathy; glomerulonephritis; alveolar hemorrhage; palpable purpura; and ANCA positivity (16). Recently, the Diagnosis and Classification criteria in Vasculitis Study (DCVAS) defined the American College of Rheumatology/European Alliance of Associations for Rheumatology (ACR/EULAR)-endorsed criteria for the classification of small-and medium-sized vessel vasculitis, including EGPA (2). These criteria consist of positively scored parameters that increase the likelihood of EGPA diagnosis, such as a maximum eosinophil count ≥1 × 10^9^/L (+5 points), nasal polyps (+3), obstructive airway disease (+3), extravascular eosinophilic-predominant inflammation (+2), and motor neuropathy and/or mononeuritis multiplex not caused by radiculopathy (+1). In contrast, there are certain parameters that, when negatively scored, reduce the likelihood of EGPA. These include a cytoplasmic ANCA (C-ANCA) pattern on immunofluorescence or positive anti-proteinase 3 (PR3)-ANCA (−3), as well as the presence of hematuria (−1). If the cumulative score reaches 6 or more, a patient with a diagnosis of small-or medium-sized vessel vasculitis can be classified as having EGPA. This scoring system achieved a sensitivity of 85% and a specificity of 99% (2).

When investigating a patient suspected of having EGPA, it is crucial to both exclude known causes of eosinophilia and ascertain the presence of a vasculitic process (11, 17). EGPA can be differentially diagnosed from several other diseases, including:

- Other forms of AAV, such as MPA and GPA: while these diseases may share some clinical and histological features, asthma and eosinophilia (especially higher than 1,500 cells/mm^3^) are not commonly present (11).
- Hyper-eosinophilic syndrome (HES), which is characterized by a sustained peripheral blood eosinophilia exceeding 1,500 cells/mm^3^ on at least two examinations, occurring over a minimum interval of 1 month. The eosinophilia is responsible for the development of organ dysfunction and/or damage (18). The organs affected in EGPA and HES are similar, and cardiac disease is a significant cause of mortality in both conditions. However, unlike EGPA, asthma is typically not present in HES, although bronchial hyperactivity may be observed. Histologically, tissue infiltration by eosinophils and fibrosis can be seen in the later stages of HES. Biopsy specimens do not exhibit signs of vasculitis. The diagnosis of HES can be facilitated by the widespread use of molecular biology techniques, as specific mutations have been identified in certain subsets of this syndrome (11).
- Allergic bronchopulmonary aspergillosis and chronic eosinophilic pneumonia, which typically present with asthma, eosinophilia, sinusitis, and lung infiltrates. However, they typically lack the extrapulmonary involvement (11).

In summary, the diagnosis of EGPA is primarily based on clinical evaluation. The presence of asthma, rhinitis or sinusitis, along with peripheral eosinophilia and symptoms suggestive of vasculitis, supports the diagnosis. However, when feasible, obtaining a tissue biopsy is recommended to further confirm the diagnosis.

### 1.1. EGPA pathogenesis

The etiology of EGPA is unknown, but it is probably driven by environmental and genetic factors (12). Exposure to silica, organic solvents, and farming activities has been linked to an increased risk of developing EGPA. On the other hand, cigarette smoking seems to be protective (19). Interesting perspectives on EGPA pathogenesis are emerging from genome-wide association studies. A recent study by Lyons et al. (20) found that the ANCA-positive and ANCA-negative phenotypes display different genetic predisposition. In fact, ANCA-positive EGPA is associated with HLA-DQ, while genetic variants involved in mucosal responses and eosinophil biology, such as GPA33 and IL5, are associated with ANCA-negative EGPA. Genetic variations in GATA3, TSLP, LPP, and BACH2 have been identified as potential contributors to the development of eosinophilic inflammation in total EGPA (20).

Also, the immunopathogenesis of EGPA seems to be related to the two phenotypes. CD4^+^ T cells polarized toward a T helper 2 (Th2) phenotype orchestrate the adaptive immune response and enhance eosinophilic reactions through IL-5 secretion (12). IL-5 plays a pivotal role in promoting eosinophil differentiation and maturation while concurrently inhibiting eosinophil apoptosis (21). This mechanism may subtend the eosinophilic features, in which eosinophils are essential to mediate tissue damage. In fact, activated eosinophils unleash their proinflammatory potential by releasing cytotoxic granules content, as major basic protein (MBP), eosinophil cationic protein (ECP), eosinophil peroxidase (EPO), and eosinophil-derived neurotoxin (EDN), and lipid mediators, thereby initiating tissue damage and provoking inflammatory responses (21, 22). This hypothesis is supported by the efficacy in EGPA patients of mepolizumab, an Anti-IL-5 therapy (16, 23). Altered Th2 immunity is also likely associated with an increased production of IgG4, which is a common feature of EGPA (24, 25).

T helper 1 (Th1) and T helper 17 cells could potentially play a role in the development of vasculitis and the formation of granulomas instead (8, 26). These cells release inflammatory cytokines such as tumor necrosis factor-α and IL-1, which triggers neutrophil priming. This, in turn, results in the subsequent translocation of the enzyme myeloperoxidase (MPO), and much less frequently proteinase 3 (PR3), from intracytoplasmic granules to the cell surface. In this setting also B cells have a pathogenic role, mediated by ANCA. In fact, neutrophils are further activated by ANCA, which can combine with their specific antigens (MPO and PR3) (27). This concept is supported by the good results obtained with rituximab (RTX), a B-cell depleting agent (28, 29). Ultimately, activated neutrophils adhere to vascular endothelial cells and subsequently migrate to the vascular wall, where they accumulate, generate reactive oxygen species free radicals, and trigger cell apoptosis and tissue damage. This cascade of events culminates in the inflammatory destruction of vascular endothelial cells and significant tissue injury (27, 30, 31).

**Figure 1:**
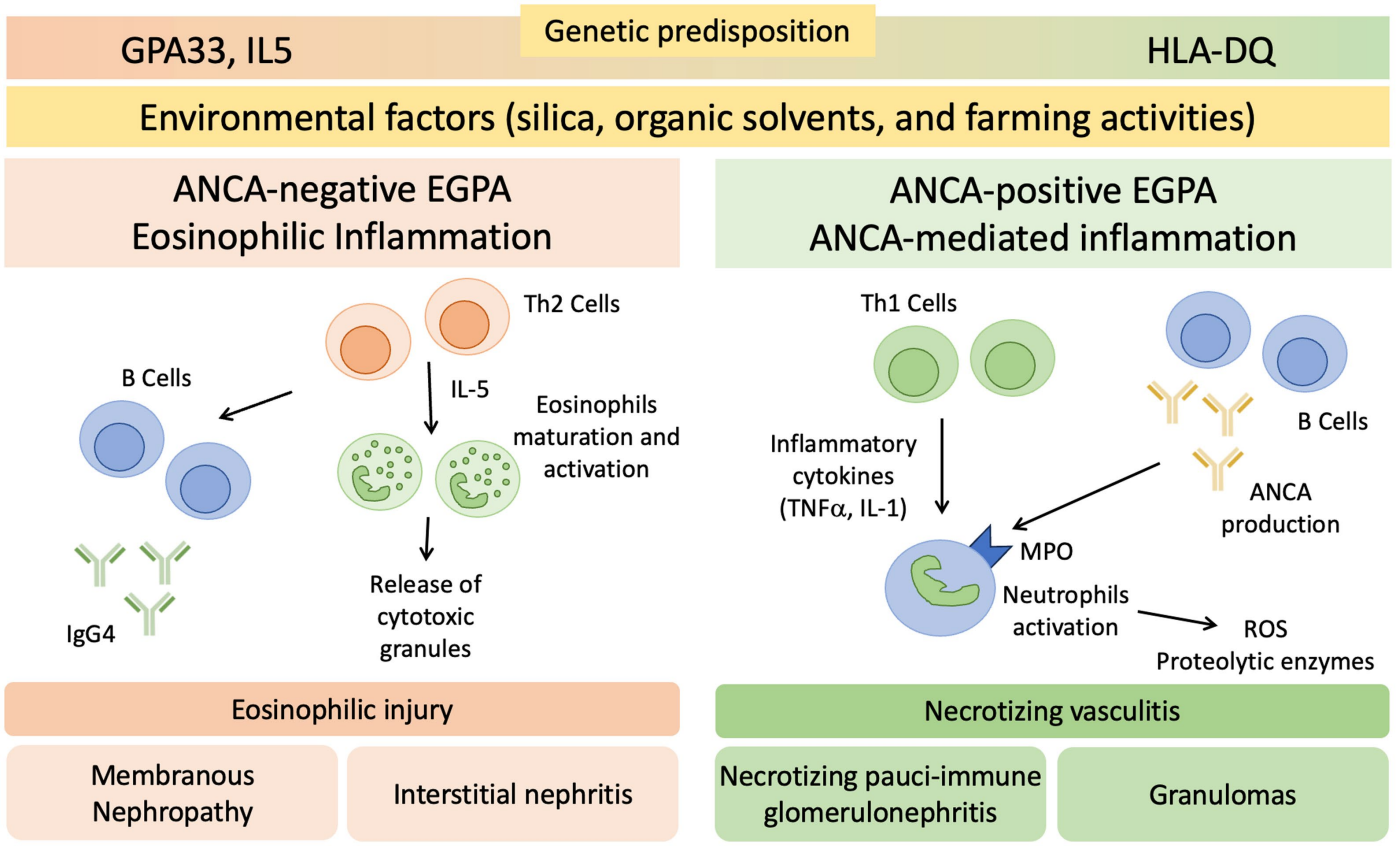
Proposed Pathogenesis of Renal Involvement in EGPA. EGPA pathogenesis is driven by environmental and genetic factors. GPA33 and IL5 are linked to ANCA-negative EGPA, HLA-DQ to ANCA-positive phenotype. CD4+ T cells polarized toward a T helper 2 (Th2) phenotype orchestrate the adaptive immune response and enhance eosinophilic reactions through IL-5 secretion. This mechanism may determine the eosinophilic feature, in which eosinophils are essential to mediate tissue damage. B cells may be involved through IgG4 production, with possible overlap with IgG4 related disease. T helper 1 (Th1), T helper 17 (Th17), and B cells potentially play a role in the development of vasculitis and the formation of granulomas. ANCA, anti-neutrophil cytoplasmic antibodies; MPO, Myeloperoxidase; ROS, reactive oxygen species; TNFα, tumor necrosis factor-α.

While the respiratory system is commonly affected, renal involvement in EGPA is not a central feature (32). The pathogenesis of renal involvement in EGPA is still not completely understood and is probably multifactorial (Figure 1). Since renal disease in EGPA is associated with ANCA positivity, it is likely that kidney damage is partly mediated by ANCA antibodies, which cause endothelial damage, inflammation and subsequent renal injury through neutrophils activation (6, 32, 33). Eosinophil-mediated mechanisms also play a role in renal involvement, as suggested by the frequent and significant interstitial infiltration, sometimes as pure acute interstitial nephritis, that is possible to observe in renal biopsies of EGPA patients (33, 34).

## 2. Renal involvement in EGPA

**Figure 2:**
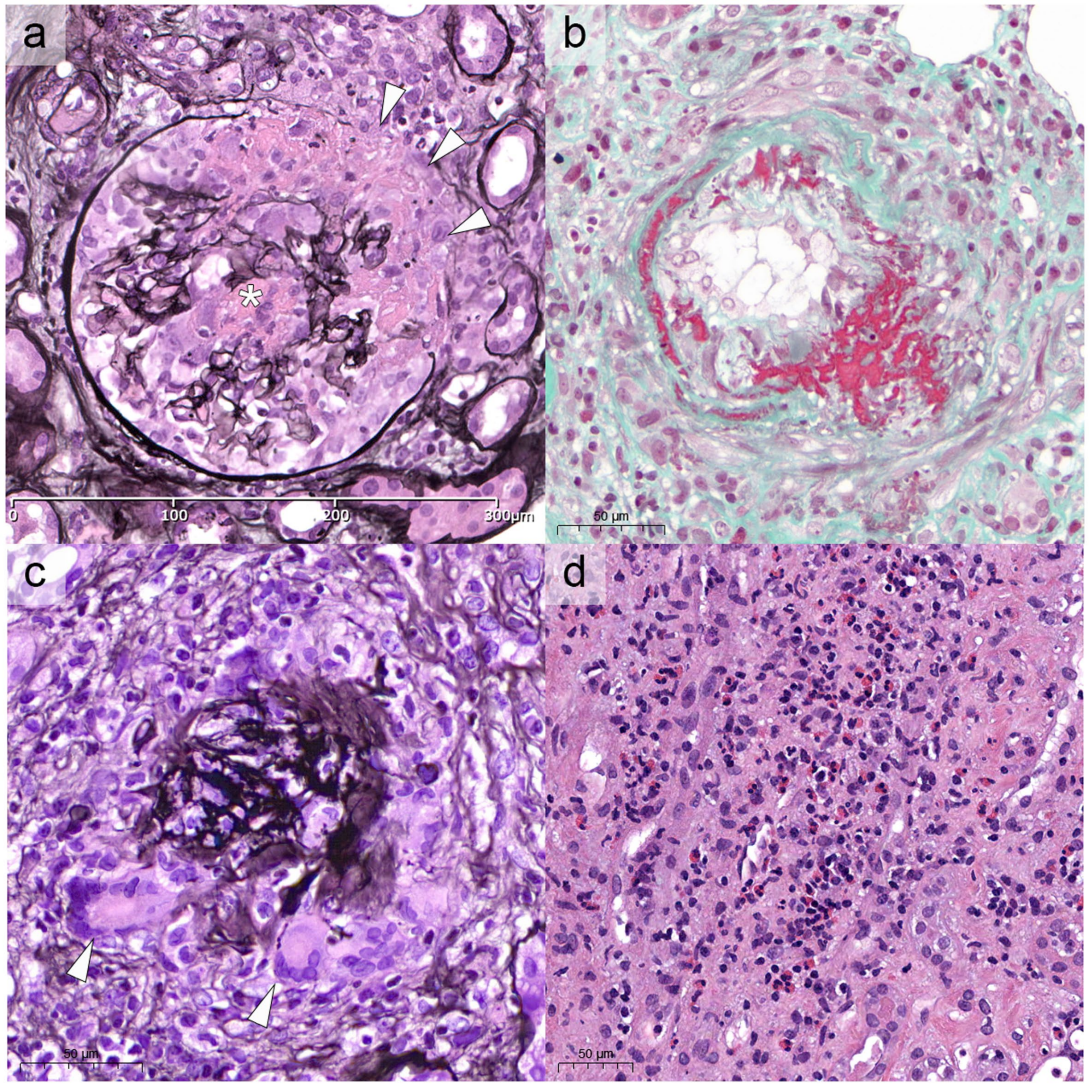
Renal histological modifications during EGPA. In **(A)** the most frequent renal manifestation of EGPA, paucimmune crescentic glomerulonephritis, with the formation of a cellular crescent, associated with fibrinoid necrosis (white asterisk) and Bowman capsule rupture (white arrowheads, Jones methenamine silver stain, x40). **(B)** Rarely, EGPA can show necrotizing, transmural arteritis involving small or medium sized arteries, as shown in Masson trichrome stain (x40). **(C)** Occasional interstitial giant cell reaction or non-necrotizing granulomas can be identified (white arrowheads), especially associated with Bowman capsule ruptures (Jones, x40). **(D)** Rarely, especially in ANCA-negative cases, the histology can be exclusively characterized by tubule-interstitial nephritis with a predominant eosinophilic infiltrate, reaching >50 elements/HPF (Hematoxylin and Eosin, x40). HPF, high power field.

**Figure 3:**
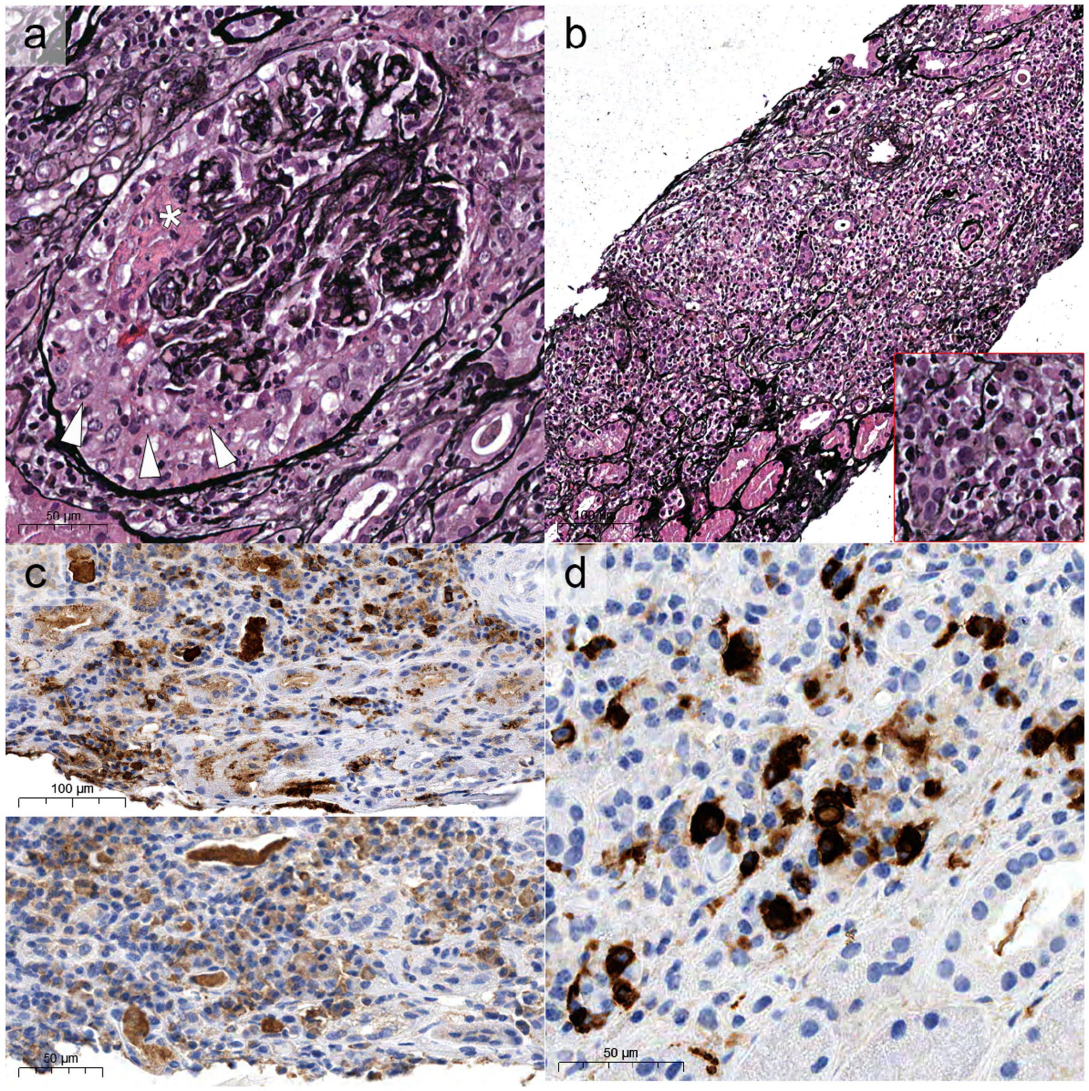
Histological overlap between EGPA and IgG4-related disease. Some cases can show “classic” aspects of ANCA-associated glomerulonephritis **(A)** with formation of crescents (white arrowheads) and fibrinoid necrosis (white asterisk, Jones, x40), associated with diffuse tubule-interstitial nephritis [**(B)**, x20] enriched with plasma cells (bottom right red inset). These cases generally show polytypic expression of lambda [**(C)**, top half of the picture] and kappa (bottom half) light chains, but with increased density of IgG4-restricted plasma cells **(D)**.

Compared to GPA and MPA, where kidney involvement is prevalent (70 and 95% of cases, respectively), only a minority (25%) of patients with EGPA will develop renal disease (6, 34, 35), more often associated with the ANCA-positive phenotype. In a retrospective study on 116 patients with EGPA, ANCA positivity was more frequently observed in cases with renal involvement (75% vs. 25.7%) (34), reaching peaks of 84% in ANCA+ cases (33). The association of ANCA positivity with glomerulonephritis in EGPA is so well established that in the 2012 Revised International Chapel Hill Consensus Conference Nomenclature of Vasculitides the sentence “ANCA is more frequent in EGPA when glomerulonephritis is present” has been added to EGPA definition (14). In a retrospective work of 31 patients with EGPA, one half presented with urinary abnormalities and the other one with RPGN (34). However, RPGN is often observed in cases of renal involvement (6, 33, 34, 36). Thus, clinical presentation of EGPA cases with renal involvement can be protean, ranging from urinary abnormalities to acute kidney injury (AKI) and rapidly progressive glomerulonephritis (RPGN) (Table 1), stressing the need for renal biopsy to confirm and characterize the specific kidney modifications (32, 37). As for GPA and MPA, the most frequent histological manifestation of EGPA is necrotizing pauci-immune glomerulonephritis (Figure 2A) (33, 34, 36), with up to 78% of AKI in EGPA showing this pattern of injury (33). As in the other ANCA-associated glomerulonephritis, the progressive formation of crescents eventually leads to Bowman capsule rupture, which have been recently demonstrated to better stratify the prognosis of these patients if associated with more widely used Berden classification and Renal Risk Score (38, 39). Even if rare, cases of EGPA with positive ANCA can present with necrotizing and transmural arteritis of small and/or medium sized arteries at renal biopsy (Figure 2B), which recently showed to have a worse prognostic significance (40, 41). Most EGPA patients with RPGN display a focal or a crescentic histological class according to Berden et al. (33, 42), suggesting that renal involvement is usually detected early in this setting compared to MPA patients with anti-MPO antibodies, in which renal involvement is diagnosed at advanced stages. This is probably determined by the severity of extra-renal symptoms, which lead to a rapid diagnosis of systemic vasculitis. As compared to the ANCA+ forms, ANCA-negative patients may show alternative patterns of injury, ranging from membranous nephropathy (MN) (10%) to membranoproliferative glomerulonephritis (3%) or acute interstitial nephritis (10%), rarely with giant cell reaction and/or interstitial non-necrotizing granulomas (Figure 2C), or more frequently with an eosinophilic predominance (Figure 2D) (33). Prominent interstitial eosinophilic infiltration is frequently present together with pauci-immune RPGN in almost half of cases, showing pathogenetic differences as compared to the interstitial inflammation seen in GPA and MPA (33). In particular, the preferential activation of T helper 2 (Th2) phenotype orchestrates the adaptive immune response and enhances eosinophilic reactions through IL-5 secretion and GPA33 as a more frequent pathway in ANCA-negative EGPA. This mechanism may determine the eosinophilic feature, in which eosinophils are essential to mediate tissue damage (20). On the other hand, up to 10% of cases can show MN as the sole renal manifestation of EGPA (33), mainly in ANCA-negative cases, showing some common genetic background with HLA alleles found in other MN forms (43). Furthermore, the presence of an overlap syndrome between AAV and IgG4-related disease has been reported, being both MN and IgG4+ plasma cell rich interstitial nephritis manifestations of IgG4-related disease (Figure 3) (24, 44). Hence, MN and ANCA-negative EGPA association may be not casual, but further studies are required to explore this hypothesis. In addition to glomerular diseases, obstructive uropathy due to ureteral involvement has been occasionally reported.

**Table 1:** Characteristics of patients with EGPA and renal involvement from the studies of Sinico et al. (34), Chen et al. (36), and Durel et al. (33).

	Sinico et al. (34) *n* = 30	Chen et al. (36) *n* = 14	Durel et al. (33) *n* = 63
Female, *n* (%)	17 (57)	8 (57)	27 (43)
Age, median (range)	57 (25–85)	53 (20–70)	63 (18–83)
BVAS, median (range)	26 (13–40)	n.d.	23 (10–39)
Extra-renal symptoms, *n* (%)			
Constitutional symptoms	27 (90)	6 (43)	48 (76)
Upper respiratory tract	n.d.	8 (57)	n.d.
Asthma	29 (97)	n.d.	63 (100)
Sinusitis	26 (87)	n.d.	44 (70)
Nasal polyps	n.d.	n.d.	23 (37)
Articular manifestations	n.d.	2 (14)	33 (52)
Peripheral nervous system involvement	18 (60)	3 (21)	29 (46)
Cutaneous involvement	18 (60)	3 (21)	25 (40)
Pulmonary involvement	14 (47)	11 (79)	n.d
Pulmonary infiltrates	n.d.	5 (36)	24 (38)
Alveolar hemorrhage	n.d.	6 (43)	10 (16)
Cardiac involvement	5 (17)	1 (7)	9 (14)
Positive ANCA serology, *n* (%)	21 (75)	14 (100)	53 (84)
PR3-ANCA	3 (10)	2 (14)	5 (9)
MPO-ANCA	18 (60)	12 (86)	44 (83)
Renal presentation			
Acute renal insufficiency, *n* (%)	15 (50)	14 (100)	47 (75)
Initial creatinine, median (range), mg/dL	1.4 (0.7–7.5)	6.0 (1.5–11.0)	2.0 (0.9–12.1)
Proteinuria, *n* (%)	26 (87)	13 (93)	60 (95)
Median proteinuria, median (range), g/day	0.5 (0.1–4.0)	1.1 (0.1–12.3)	1.0 (0.2–17.0)
Microscopic hematuria, *n* (%)	30 (100)	n.d.	53 (84)
Renal histology, *n* (%)			
Pauci-immune necrotizing GN and acute interstitial nephritis	2 (14)	10 (71)	28 (44)
Isolated pauci-immune necrotizing GN	9 (64)	3 (21)	21 (33)
Isolated acute interstitial nephritis	1 (7)	1 (7)	6 (9)
Membranous nephropathy	0 (0)	0 (0)	6 (9)
Membranoproliferative GN	0 (0)	0 (0)	2 (3)

## 3. Treatment

**Figure 4:**
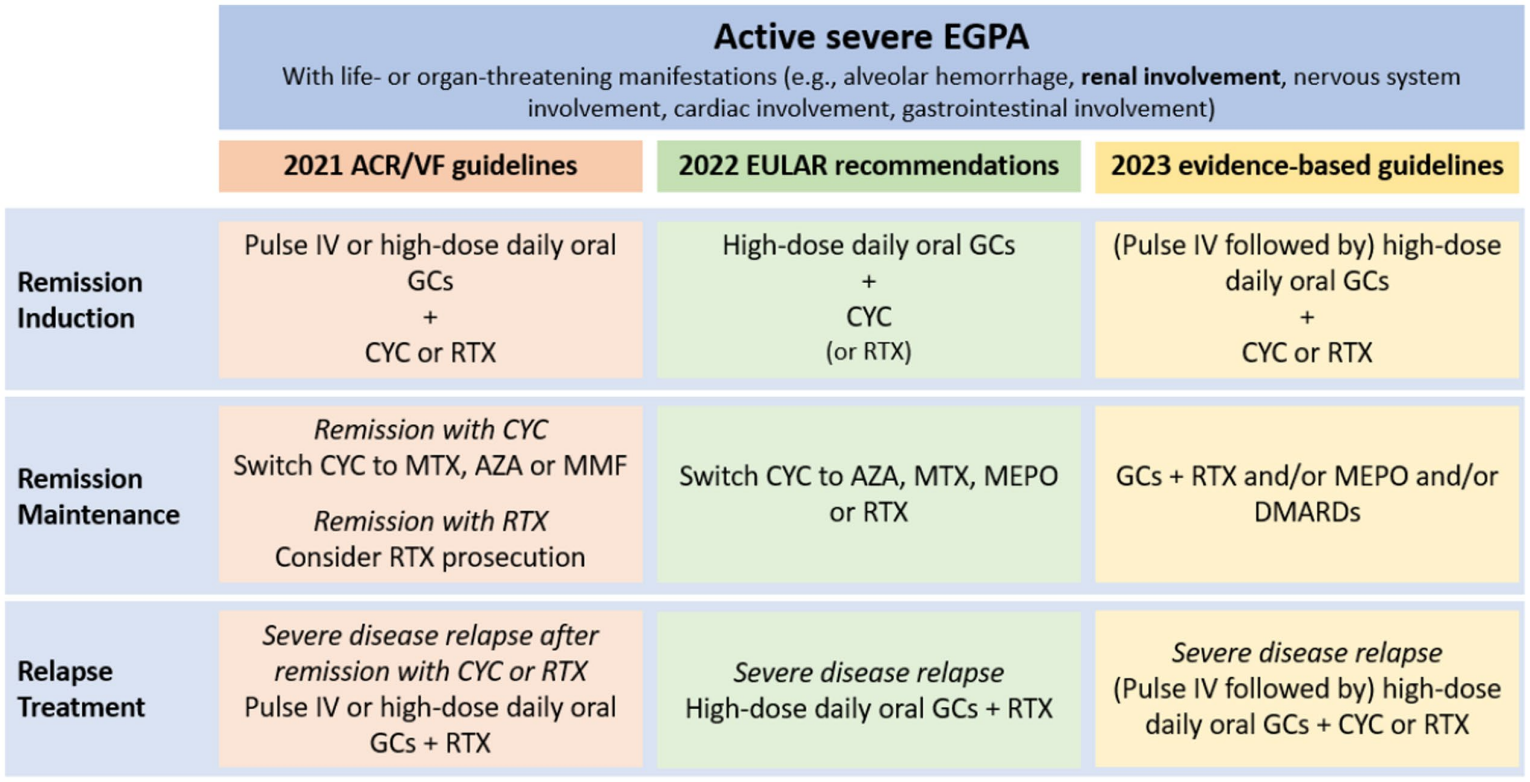
Schematic representation of severe EGPA treatment according to the three main guidelines (12, 48, 49). IV pulses = IV methylprednisolone 500–1,000 mg/day for 3–5 days (total cumulative dose 3 g). High dose oral GCs = prednisone 0.75–1 mg/kg/day (up to 80 mg/day). ACR/VF, American College of Rheumatology/Vasculitis Foundation; AZA, azathioprine; CYC, cyclophosphamide; DMARDs, disease-modifying antirheumatic drugs; EULAR, indications from the three main references for the treatment of EGPA are summarized; GCs, glucocorticoids; IV, intravenous; MEPO, mepolizumab; MTX, methotrexate; RTX, rituximab.

The treatment of EGPA is based on remission-induction and remission-maintenance (45–47). Remission-induction treatment should be tailored to disease severity, defined according to the presence at the diagnosis of at least one adverse prognostic factor included in the Five-Factor Score (FFS) (12). The FFS is a prognostic tool used to assess the risk of mortality in patients diagnosed with EGPA and includes: renal insufficiency defined as serum creatinine >1.58 mg/dL, proteinuria exceeding 1 g per day, cardiomyopathy, gastrointestinal involvement and central nervous system involvement (44). FFS was revised in 2011 by Guillevin et al., which added age > 65 years to cardiomyopathy, gastrointestinal involvement, and renal insufficiency (creatinine ≥1.7 mg/dL), eliminating central nervous system involvement. Independently from which score is chosen, renal involvement is sufficient to define a severe disease and drive a more aggressive treatment. In Figure 4, the indications from the three main references for the treatment of EGPA are summarized (12, 48, 49). Patients with severe disease are recommended to receive pulsed intravenous glucocorticoids (GCs) (typically daily methylprednisolone pulses of 500–1,000 mg each over 3 days, for a maximum total dose of 3 g), followed by high-dose oral GCs (0.75–1 mg/kg per day) (12, 49). In severe disease cyclophosphamide (CYC) should be added to GCs for remission induction (49). In a randomized controlled trial (RCT) on 48 EGPA patients with FFS ≥ 1, of which 19 with renal involvement, a lower rate of minor relapses was observed after 12 CYC pulses than after 6 CYC pulses (administered every 2 weeks for 1 month, then every 4 weeks thereafter, at a dose of 0.6 g/m^2^ per pulse) (50). However, 12 CYC pulses did not improved response rate or reduced severe relapses (50). Therefore, there is no consensus on the optimal duration of CYC therapy, which must be balanced between the efficacy and the potentially harmful dose-related side effects (51). The latest EULAR guidelines recommend switching to a less intensive remission maintenance therapy after 6 pulses of CYC if remission is achieved (49). In cases where patients show slow improvement but do not achieve complete remission within 6 months, longer induction periods with CYC may be considered, extending up to 9–12 months (12).

Data from observational studies suggested RTX as a potential alternative for remission induction (28, 52–54). The RCT REOVAS, presented as abstract at the 2021 American College of Rheumatology convergence, found that in EGPA patients with FFS ≥ 1 RTX (two 1-gram pulses on day 1 and 15) is comparable with CYC (nine intravenous pulses over 13 weeks) for induction of remission (defined as Birmingham Vasculitis Activity Score [BVAS] of zero and a prednisone dose ≤7.5 mg per day). The two groups showed comparable adverse events, cumulative prednisone doses, and quality of life (55). Differently from previous observational studies (28, 54), the response to RTX was similar in ANCA-positive and ANCA-negative patients (55). Hence, this study suggests that RTX may be an alternative to CYC and that EGPA treatment should not be influenced solely by ANCA status (49). Nevertheless, the REOVAS trial was structured as a superiority trial. Given that the primary endpoint of on-treatment remission was attained at comparable rates in both cohorts, there exists insufficient substantial evidence to definitively establish non-inferiority (55).

There are no data on GCs tapering strategies in EGPA. Therefore, evidence obtained from the PEXIVAS trial on GCs tapering in GPA and MPA should be used as orientation (56). The goal is to achieve through a stepwise reduction a dose of 5 mg prednisolone equivalent per day by 4–5 months (49). The future goal for treatment strategies is to spare GCs. In this perspective, mepolizumab may be an interesting add-on therapy. The MIRRA RCT, which investigated the efficacy and safety of mepolizumab compared to placebo in achieving remission in EGPA patients without organ-or life-threatening manifestations, demonstrated a higher percentage of remission and more weeks in remission in patients treated with mepolizumab, allowing a reduction in the GCs cumulative dose (16). Some case reports described the effectiveness of mepolizumab as an add-on therapy to induce remission, in particular in the case of pure interstitial nephritis (57–59). Mepolizumab was given in addition to GCs and CYC (59), or to GCs alone (57, 58). In another case report, the addition of mepolizumab to GCs and RTX achieved successful treatment of a case of EGPA with multiorgan involvement, notably including renal damage determined by necrotizing pauci-immune glomerulonephritis and severe interstitial nephritis (60). The hypothesis that a synergistic treatment with complementary mechanisms of action, such as the combination of RTX and MEPO, could enhance the remission rates of EGPA components, finds further validation in the outcomes of the European multicenter retrospective observational study conducted by Bettiol et al. (61) This study demonstrated the efficacy of sequential RTX and mepolizumab therapy in inducing and sustaining remission for both systemic and respiratory EGPA symptoms. Additionally, two other anti-IL-5 therapies, reslizumab and benralizumab, have undergone investigation, yielding promising results as GCs sparing agents in phase 2 open-label trials involving a limited number of patients (62, 63). In theory, these drugs should be more effective in ANCA-negative individuals who exhibit a profile with eosinophilic features. However, this hypothesis has not yet been substantiated by clinical data (64, 65). Currently, the efficacy and safety of benralizumab are under evaluation, comparing it to mepolizumab in EGPA patients who are receiving standard care therapy (NCT 04157348). Another noteworthy anti-IL-5 agent, notable for a long-acting properties, currently under investigation is depemokimab (NCT05263934) (66). However, further studies are needed to evaluate the actual role of mepolizumab and other anti-IL-5 agents in the induction therapy of EGPA with renal involvement.

After remission-induction, remission-maintenance is necessary to avoid relapses, but also to reduce the risk of drug-related toxicity. However, data regarding patients with severe EGPA are scant and not exhaustive. Observational studies failed to prove a better relapse-free survival with azathioprine (AZA), methotrexate (MTX) and leflunomide combined with GCs, compared to GCs alone (67, 68). However, these drugs are often used in clinical practice during the maintenance phase (12, 48, 69). RTX seems to be effective also for remission maintenance. In an observational study on 21 patients with EGPA, scheduled maintenance RTX (500 mg every 6 months) reduced the relapse rate compared to single gram infusion administered in case of relapse (29). The use of CYC for remission maintenance is not indicated because of its toxicity (49). Moreover, in a single-center prospective RCT that investigated the use of oral CYC versus MTX for 1 year following remission induction with CYC in different subtypes of AAV, no significant difference in relapse rates was observed between the two treatment arms in the subgroup of 30 patients with severe EGPA (70).

The treatment of severe systemic relapses is based on RTX or CYC. RTX is preferred when re-treatment with CYC is contraindicated, or in patients who previously achieved remission on RTX or failed to respond to CYC. CYC should be considered in cases of severe or life-threatening complications and/or in patients who have previously failed to respond to RTX (12, 28, 29, 52, 70).

It’s now recognized that ANCA status describes two different phenotypes of EGPA, characterized by different clinical presentation, and probably also pathogenesis. However, there is no evidence that different ANCA phenotypes necessitate different therapeutic approaches (12). Preliminary evidence suggested that ANCA-positive patients may be more susceptible to RTX (28, 54), but these results have been questioned by the REOVAS trial (55). In the context of ANCA-negative cases linked to Th2-related etiology, anti-IL5 therapies are emerging as a promising approach (71). Nevertheless, clinical data remains relatively limited, and another potentially effective biologic, omalizumab, an anti-IgE monoclonal antibody, has exhibited efficacy in asthma treatment but not in extrapulmonary manifestations (72, 73). Hence, the prospect of selectively targeting eosinophilic or vasculitic inflammation undeniably represents a groundbreaking approach. However, to successfully integrate these novel treatments into the landscape of this complex disease, additional clinical data is required, and significant progress must be made in understanding the etiopathogenesis of renal involvement in EGPA.

## 4. Outcome and prognosis

The presence of kidney involvement in AAV determines higher morbidity and mortality rates (7, 74). However, EGPA patients with renal involvement have favorable outcomes when treated with GCs and immunosuppressants. Compared to GPA and MPA, patients’ survival is good (34, 75–79). In the retrospective work by Sinico et al., the five-year mortality rate in EGPA patients with renal abnormalities was 11.7%. In the same study, favorable renal survival outcomes were reported. Only one patient reached end-stage renal disease (ESRD), and none of the patients experienced a doubling of serum creatinine levels after a mean follow-up of approximately 5 years (34). In the study by Durel et al., after a median follow-up of 48 months 92% of patients were alive and 17% (11 patients) reached ESRD, with nine patients (14%) on chronic dialysis and two (3%) who underwent kidney transplantation (33). In another retrospective study, out of twelve patients monitored over a median duration of 43.5 months, two patients ESRD, nine exhibited chronic kidney disease with an estimated glomerular filtration rate (eGFR) < 60 mL/min, and two patients maintained a normal eGFR (36). ANCA status may have prognostic implications since overall survival seems to be worse in ANCA-negative patients, while relapses do appear to occur more frequently in ANCA-positive patients, even if some controversies exist (6, 9). The worse prognosis of ANCA negative patients is probably caused by the higher frequency of cardiac involvement (12).

## 5. Conclusion

EGPA is a rare form of vasculitis, and renal involvement is present in just 25% of cases. However, kidney disease is an adverse prognostic factor and may prompt a more aggressive treatment based on GCs and immunosuppressants. At the moment, the management of EGPA and kidney disease in EGPA is challenging, as it remains a condition that is not easily diagnosed and without treatments validated by clinical trials. RCT are necessary to fill this gap and to test current and newer biological therapies. Moreover, other unresolved challenges remain for the future, as understanding better the pathogenesis and the role of genetics, and if these are truly associated with the two phenotypes based on ANCA status. This will open new perspectives on the treatment of EGPA, allowing the development of therapies tailored to the different EGPA subsets.

## Author contributions

FR and RS: conceptualization. FR, MC, and RS: data creation. FR, VL’M, and RS: writing. FR, VL’M, and FP: visualization. FR, VL’M, MC, FP, and RS: review editing. All authors contributed to the article and approved the submitted version.

## Conflict of interest

The authors declare that the research was conducted in the absence of any commercial or financial relationships that could be construed as a potential conflict of interest.

## Publisher’s note

All claims expressed in this article are solely those of the authors and do not necessarily represent those of their affiliated organizations, or those of the publisher, the editors and the reviewers. Any product that may be evaluated in this article, or claim that may be made by its manufacturer, is not guaranteed or endorsed by the publisher.

## References

[1] White J Dubey S. Eosinophilic granulomatosis with polyangiitis: a review. Autoimmun Rev. (2023) 22:103219. doi: 10.1016/j.autrev.2022.103219, PMID: 36283646

[2] Grayson PC Ponte C Suppiah R Robson JC Craven A Judge A. 2022 American College of Rheumatology/European Alliance of associations for rheumatology classification criteria for eosinophilic granulomatosis with polyangiitis. Ann Rheum Dis. (2022) 81:309–14. doi: 10.1136/annrheumdis-2021-221794, PMID: 35110334

[3] Villa-Forte A. Eosinophilic granulomatosis with polyangiitis. Postgrad Med. (2023) 135:52–60. doi: 10.1080/00325481.2022.2134624, PMID: 36259957

[4] Romero Gómez C Hernández Negrín H Ayala Gutiérrez MDM. Eosinophilic granulomatosis with polyangiitis. Med Clin (Barc). (2023) 160:310–7. doi: 10.1016/j.medcli.2023.01.003, PMID: 36774291

[5] Trivioli G Terrier B Vaglio A. Eosinophilic granulomatosis with polyangiitis: understanding the disease and its management. Rheumatology. (2020) 59:iii84–94. doi: 10.1093/rheumatology/kez570, PMID: 32348510

[6] Comarmond C Pagnoux C Khellaf M Cordier J-F Hamidou M Viallard J-F. Eosinophilic granulomatosis with polyangiitis (Churg-Strauss): clinical characteristics and long-term followup of the 383 patients enrolled in the French Vasculitis study group cohort. Arthritis Rheum. (2013) 65:270–81. doi: 10.1002/art.37721, PMID: 23044708

[7] Sinico RA Di Toma L Maggiore U Bottero P Radice A Tosoni C. Prevalence and clinical significance of antineutrophil cytoplasmic antibodies in Churg-Strauss syndrome. Arthritis Rheum. (2005) 52:2926–35. doi: 10.1002/art.21250, PMID: 16142760

[8] Vaglio A Buzio C Zwerina J. Eosinophilic granulomatosis with polyangiitis (Churg-Strauss): state of the art. Allergy. (2013) 68:261–73. doi: 10.1111/all.12088, PMID: 23330816

[9] Healy B Bibby S Steele R Weatherall M Nelson H Beasley R. Antineutrophil cytoplasmic autoantibodies and myeloperoxidase autoantibodies in clinical expression of Churg-Strauss syndrome. J Allergy Clin Immunol. (2013) 131:571–6.e1–6. doi: 10.1016/j.jaci.2012.05.058 22920496

[10] Sablé-Fourtassou R Cohen P Mahr A Pagnoux C Mouthon L Jayne D. Antineutrophil cytoplasmic antibodies and the Churg-Strauss syndrome. Ann Intern Med. (2005) 143:632–8. doi: 10.7326/0003-4819-143-9-200511010-00006, PMID: 16263885

[11] Sinico RA Bottero P. Churg-Strauss angiitis. Best Pract Res Clin Rheumatol. (2009) 23:355–66. doi: 10.1016/j.berh.2009.02.004, PMID: 19508943

[12] Emmi G Bettiol A Gelain E Bajema IM Berti A Burns S. Evidence-based guideline for the diagnosis and management of eosinophilic granulomatosis with polyangiitis. Nat Rev Rheumatol. (2023) 19:378–93. doi: 10.1038/s41584-023-00958-w, PMID: 37161084

[13] Jennette JC Falk RJ Bacon PA Basu N Cid MC Ferrario F. 2012 revised international Chapel Hill consensus conference nomenclature of Vasculitides. Arthritis Rheum. (2013) 65:1–11. doi: 10.1002/art.37715, PMID: 23045170

[14] Masi AT Hunder GG Lie JT Michel BA Bloch DA Arend WP. The American College of Rheumatology 1990 criteria for the classification of Churg-Strauss syndrome (allergic granulomatosis and angiitis). Arthritis Rheum. (1990) 33:1094–100. doi: 10.1002/art.1780330806, PMID: 2202307

[15] Lanham JG Elkon KB Pusey CD Hughes GR. Systemic vasculitis with asthma and eosinophilia: a clinical approach to the Churg-Strauss syndrome. Medicine (Baltimore). (1984) 63:65–81. doi: 10.1097/00005792-198403000-00001, PMID: 6366453

[16] Wechsler ME Akuthota P Jayne D Khoury P Klion A Langford CA. Mepolizumab or placebo for eosinophilic granulomatosis with polyangiitis. N Engl J Med. (2017) 376:1921–32. doi: 10.1056/NEJMoa1702079, PMID: 28514601 PMC5548295

[17] Zhao B Zheng H Yang T Zheng R. Eosinophilic granulomatosis with polyangiitis in allergic asthma: efforts to make early diagnosis possible. Allergy Asthma Proc. (2023) 44:59–63. doi: 10.2500/aap.2023.44.220072, PMID: 36719697

[18] Radin M Bertero L Roccatello D Sciascia S. Severe multi-organ failure and Hypereosinophilia: when to call it “idiopathic”? J Investig Med High Impact Case Rep. (2018) 6:2324709618758347. doi: 10.1177/2324709618758347, PMID: 29479541 PMC5818087

[19] Maritati F Peyronel F Fenaroli P Pegoraro F Lastrucci V Benigno GD. Occupational exposures and smoking in eosinophilic granulomatosis with polyangiitis: a case-control study. Arthritis Rheumatol Hoboken NJ. (2021) 73:1694–702. doi: 10.1002/art.41722, PMID: 33750006

[20] Lyons PA Peters JE Alberici F Liley J Coulson RMR Astle W. Genome-wide association study of eosinophilic granulomatosis with polyangiitis reveals genomic loci stratified by ANCA status. Nat Commun. (2019) 10:5120. doi: 10.1038/s41467-019-12515-9 31719529 PMC6851141

[21] Furuta S Iwamoto T Nakajima H. Update on eosinophilic granulomatosis with polyangiitis. Allergol Int. (2019) 68:430–6. doi: 10.1016/j.alit.2019.06.004, PMID: 31266709

[22] Wechsler ME Munitz A Ackerman SJ Drake MG Jackson DJ Wardlaw AJ. Eosinophils in health and disease: a state-of-the-art review. Mayo Clin Proc. (2021) 96:2694–707. doi: 10.1016/j.mayocp.2021.04.025, PMID: 34538424

[23] Bettiol A Urban ML Dagna L Cottin V Franceschini F Del Giacco S. Mepolizumab for eosinophilic granulomatosis with polyangiitis: a European Multicenter observational study. Arthritis Rheumatol Hoboken NJ. (2022) 74:295–306. doi: 10.1002/art.41943, PMID: 34347947 PMC9305132

[24] Vaglio A Strehl JD Manger B Maritati F Alberici F Beyer C. IgG4 immune response in Churg-Strauss syndrome. Ann Rheum Dis. (2012) 71:390–3. doi: 10.1136/ard.2011.155382, PMID: 22121132

[25] Kubo S Kanda R Nawata A Miyazaki Y Kawabe A Hanami K. Eosinophilic granulomatosis with polyangiitis exhibits T cell activation and IgG4 immune response in the tissue; comparison with IgG4-related disease. RMD Open. (2022) 8:e002086. doi: 10.1136/rmdopen-2021-002086, PMID: 35260476 PMC8906049

[26] Vaglio A Casazza I Grasselli C Corradi D Sinico RA Buzio C. Churg-Strauss syndrome. Kidney Int. (2009) 76:1006–11. doi: 10.1038/ki.2009.210, PMID: 19516244

[27] Ge S Zhu X Xu Q Wang J An C Hu Y. Neutrophils in ANCA-associated vasculitis: mechanisms and implications for management. Front Pharmacol. (2022) 13:957660. doi: 10.3389/fphar.2022.957660.eCollection 2022 36210838 PMC9545605

[28] Mohammad AJ Hot A Arndt F Moosig F Guerry M-J Amudala N. Rituximab for the treatment of eosinophilic granulomatosis with polyangiitis (Churg-Strauss). Ann Rheum Dis. (2016) 75:396–401. doi: 10.1136/annrheumdis-2014-206095, PMID: 25467294

[29] Emmi G Rossi GM Urban ML Silvestri E Prisco D Goldoni M. Scheduled rituximab maintenance reduces relapse rate in eosinophilic granulomatosis with polyangiitis. Ann Rheum Dis. (2018) 77:952–4. doi: 10.1136/annrheumdis-2017-211897, PMID: 28814426

[30] Al-Hussain T Hussein MH Conca W Al Mana H Akhtar M. Pathophysiology of ANCA-associated Vasculitis. Adv Anat Pathol. (2017) 24:226–34. doi: 10.1097/PAP.0000000000000154, PMID: 28537941

[31] Brilland B Garnier A-S Chevailler A Jeannin P Subra J-F Augusto J-F. Complement alternative pathway in ANCA-associated vasculitis: two decades from bench to bedside. Autoimmun Rev. (2020) 19:102424. doi: 10.1016/j.autrev.2019.102424, PMID: 31734405

[32] Doreille A Buob D Bay P Julien M Riviere F Rafat C. Renal involvement in eosinophilic granulomatosis with polyangiitis. Kidney Int Rep. (2021) 6:2718–21. doi: 10.1016/j.ekir.2021.07.002, PMID: 34622111 PMC8484126

[33] Durel C-A Sinico RA Teixeira V Jayne D Belenfant X Marchand-Adam S. Renal involvement in eosinophilic granulomatosis with polyangiitis (EGPA): a multicentric retrospective study of 63 biopsy-proven cases. Rheumatology. (2021) 60:359–65. doi: 10.1093/rheumatology/keaa416, PMID: 32856066

[34] Sinico RA Di Toma L Maggiore U Tosoni C Bottero P Sabadini E. Renal involvement in Churg-Strauss syndrome. Am J Kidney Dis Off J Natl Kidney Found. (2006) 47:770–9. doi: 10.1053/j.ajkd.2006.01.026 16632015

[35] Kronbichler A Shin JI Lee KH Nakagomi D Quintana LF Busch M. Clinical associations of renal involvement in ANCA-associated vasculitis. Autoimmun Rev. (2020) 19:102495. doi: 10.1016/j.autrev.2020.102495, PMID: 32068190

[36] Chen Y Ding Y Liu Z Zhang H Liu Z Hu W. Long-term outcomes in antineutrophil cytoplasmic autoantibody–positive eosinophilic granulomatosis with polyangiitis patients with renal involvement: a retrospective study of 14 Chinese patients. BMC Nephrol. (2016) 17:101. doi: 10.1186/s12882-016-0319-2 27461086 PMC4962371

[37] L’Imperio V Vischini G Pagni F Ferraro PM. Bowman’s capsule rupture on renal biopsy improves the outcome prediction of ANCA-associated glomerulonephritis classifications. Ann Rheum Dis. (2022) 81:e95. doi: 10.1136/annrheumdis-2020-217979, PMID: 32532749

[38] L’Imperio V Pagni F. Unveiling the role of additional histological parameters in ANCA-associated Vasculitis. J Am Soc Nephrol JASN. (2022) 33:1226–7. doi: 10.1681/ASN.2022020208, PMID: 35396263 PMC9161806

[39] L’Imperio V Vischini G Ferraro M Pagni F. Response to: “correspondence on ‘Bowman’s capsule rupture on renal biopsy improves the outcome prediction of ANCA-associated glomerulonephritis classifications’” by Hakroush and Tampe. Ann Rheum Dis. (2023) 82:e126. doi: 10.1136/annrheumdis-2021-219988, PMID: 33547064

[40] Boudhabhay I Delestre F Coutance G Gnemmi V Quemeneur T Vandenbussche C. Reappraisal of renal arteritis in ANCA-associated Vasculitis: clinical characteristics, pathology, and outcome. J Am Soc Nephrol JASN. (2021) 32:2362–74. doi: 10.1681/ASN.2020071074, PMID: 34155059 PMC8729836

[41] Berden AE Ferrario F Hagen EC Jayne DR Jennette JC Joh K. Histopathologic classification of ANCA-associated glomerulonephritis. J Am Soc Nephrol. (2010) 21:1628. doi: 10.1681/ASN.2010050477 20616173

[42] Kronbichler A Bettac EL. Kidney disease in eosinophilic granulomatosis with polyangiitis: expect the unexpected. Rheumatology. (2021) 60:1–2. doi: 10.1093/rheumatology/keaa571, PMID: 33147610

[43] Danlos F-X Rossi GM Blockmans D Emmi G Kronbichler A Durupt S. Antineutrophil cytoplasmic antibody-associated vasculitides and IgG4-related disease: a new overlap syndrome. Autoimmun Rev. (2017) 16:1036–43. doi: 10.1016/j.autrev.2017.07.020, PMID: 28780079

[44] Guillevin L Pagnoux C Seror R Mahr A Mouthon L Toumelin PL. Group (FVSG) for the FVS. The five-factor score revisited: assessment of prognoses of systemic necrotizing Vasculitides based on the French Vasculitis study group (FVSG) cohort. Medicine (Baltimore). (2011) 90:19. doi: 10.1097/MD.0b013e318205a4c6, PMID: 21200183

[45] Ford JA Aleatany Y Gewurz-Singer O. Therapeutic advances in eosinophilic granulomatosis with polyangiitis. Curr Opin Rheumatol. (2022) 34:158–64. doi: 10.1097/BOR.0000000000000873, PMID: 35440531

[46] Bloom JL Langford CA Wechsler ME. Therapeutic advances in eosinophilic granulomatosis with polyangiitis. Rheum Dis Clin N Am. (2023) 49:563–84. doi: 10.1016/j.rdc.2023.03.006, PMID: 37331733

[47] Pagnoux C Berti A. Advances in the pharmacotherapeutic management of eosinophilic granulomatosis with polyangiitis. Expert Opin Pharmacother. (2023) 24:1269–81. doi: 10.1080/14656566.2023.2216379, PMID: 37204027

[48] Chung SA Langford CA Maz M Abril A Gorelik M Guyatt G. American College of Rheumatology/Vasculitis Foundation guideline for the management of antineutrophil cytoplasmic antibody–associated vasculitis. Arthritis Care Res. (2021) 73:1088–105. doi: 10.1002/acr.24634, PMID: 34235880 PMC12344527

[49] Hellmich B Sanchez-Alamo B Schirmer JH Berti A Blockmans D Cid MC. EULAR recommendations for the management of ANCA-associated vasculitis: 2022 update. Ann Rheum Dis. (2023):ard-2022-223764. doi: 10.1136/ard-2022-223764, PMID: 36927642

[50] Cohen P Pagnoux C Mahr A Arène J-P Mouthon L Le Guern V. Churg-Strauss syndrome with poor-prognosis factors: a prospective multicenter trial comparing glucocorticoids and six or twelve cyclophosphamide pulses in forty-eight patients. Arthritis Care Res. (2007) 57:686–93. doi: 10.1002/art.22679 17471546

[51] Fraiser LH Kanekal S Kehrer JP. Cyclophosphamide toxicity. Drugs. (1991) 42:781–95. doi: 10.2165/00003495-199142050-00005, PMID: 1723374

[52] Casal Moura M Berti A Keogh KA Volcheck GW Specks U Baqir M. Asthma control in eosinophilic granulomatosis with polyangiitis treated with rituximab. Clin Rheumatol. (2020) 39:1581–90. doi: 10.1007/s10067-019-04891-w, PMID: 31897956

[53] Thiel J Troilo A Salzer U Schleyer T Halmschlag K Rizzi M. Rituximab as induction therapy in eosinophilic granulomatosis with polyangiitis refractory to conventional immunosuppressive treatment: a 36-month follow-up analysis. J Allergy Clin Immunol Pract. (2017) 5:1556–63. doi: 10.1016/j.jaip.2017.07.027, PMID: 28916432

[54] Teixeira V Mohammad AJ Jones RB Smith R Jayne D. Efficacy and safety of rituximab in the treatment of eosinophilic granulomatosis with polyangiitis. RMD Open. (2019) 5:e000905. doi: 10.1136/rmdopen-2019-000905, PMID: 31245051 PMC6560673

[55] Terrier B. Pugnet G. de Moreuil Claire Bonnotte B. Benhamou Y. Diot E.. Rituximab versus Conventional Therapeutic Strategy for Remission Induction in Eosinophilic Granulomatosis with Polyangiitis: A Double-blind, Randomized, Controlled Trial. ACR Meet Abstr. Available at: https://acrabstracts.org/abstract/rituximab-versus-conventional-therapeutic-strategy-for-remission-induction-in-eosinophilic-granulomatosis-with-polyangiitis-a-double-blind-randomized-controlled-trial/10.7326/ANNALS-24-03947 40720835

[56] Walsh M Merkel PA Peh C-A Szpirt WM Puéchal X Fujimoto S. Plasma exchange and glucocorticoids in severe ANCA-associated Vasculitis. N Engl J Med. (2020) 382:622–31. doi: 10.1056/NEJMoa1803537, PMID: 32053298 PMC7325726

[57] Hattori K Teramachi Y Kobayashi Y Ito T Morinaga T Tamai H. A case of effective mepolizumab induction therapy for severe eosinophilic granulomatosis with polyangiitis diagnosed by eosinophilic cholecystitis and interstitial nephritis. Case Rep Rheumatol. (2021) 2021:e6678893. doi: 10.1155/2021/6678893, PMID: 34239754 PMC8235979

[58] Hayama Y Tomyo F Ueno M Asakawa S Arai S Yamazaki O. Renal involvement as rare acute tubulointerstitial nephritis in a patient with eosinophilic disorder treated with early add-on Administration of Mepolizumab. Intern Med. (2021) 60:3759–64. doi: 10.2169/internalmedicine.7490-21, PMID: 34092738 PMC8710382

[59] Nawata T Shibuya M Takeshita Y Kubo M Uesugi N Yano M. Glomerulonephritis and interstitial nephritis Originating from Vasculitis of the interlobular arteries of the kidney in a patient with eosinophilic granulomatosis with polyangiitis. Case Rep Rheumatol. (2022) 2022:e9606981. doi: 10.1155/2022/9606981, PMID: 36212163 PMC9534698

[60] Afiari A Gabriel A Gaiki MR Afiari A Gabriel A Gaiki MR. Concurrent use of mepolizumab and rituximab for eosinophilic granulomatosis with polyangiitis and multisystem involvement. Cureus. (2020) 12:e9242. doi: 10.7759/cureus.9242, PMID: 32821588 PMC7430662

[61] Bettiol A Urban ML Bello F Fiori D Mattioli I Lopalco G. Sequential rituximab and mepolizumab in eosinophilic granulomatosis with polyangiitis (EGPA): a European multicentre observational study. Ann Rheum Dis. (2022) 81:1769–72. doi: 10.1136/ard-2022-222776, PMID: 35850947

[62] Manka LA Guntur VP Denson JL Dunn RM Dollin YT Strand MJ. Efficacy and safety of reslizumab in the treatment of eosinophilic granulomatosis with polyangiitis. Ann Allergy Asthma Immunol Off Publ Am Coll Allergy Asthma Immunol. (2021) 126:696–701.e1. doi: 10.1016/j.anai.2021.01.035 33548468

[63] Guntur VP Manka LA Denson JL Dunn RM Dollin YT Gill M. Benralizumab as a steroid-sparing treatment option in eosinophilic granulomatosis with polyangiitis. J Allergy Clin Immunol Pract. (2021) 9:1186–1193.e1. doi: 10.1016/j.jaip.2020.09.054, PMID: 33065367

[64] Kouverianos I Angelopoulos A Daoussis D. The role of anti-eosinophilic therapies in eosinophilic granulomatosis with polyangiitis: a systematic review. Rheumatol Int. (2023) 43:1245–52. doi: 10.1007/s00296-023-05326-1, PMID: 37085573 PMC10185576

[65] Koike H Nishi R Yagi S Furukawa S Fukami Y Iijima M. A review of anti-IL-5 therapies for eosinophilic granulomatosis with polyangiitis. Adv Ther. (2023) 40:25–40. doi: 10.1007/s12325-022-02307-x, PMID: 36152266

[66] Fijolek J Radzikowska E. Eosinophilic granulomatosis with polyangiitis - advances in pathogenesis, diagnosis, and treatment. Front Med. (2023) 10:1145257. doi: 10.3389/fmed.2023.1145257 PMC10193253 37215720

[67] Moosig F Bremer JP Hellmich B Holle JU Holl-Ulrich K Laudien M. A vasculitis Centre based management strategy leads to improved outcome in eosinophilic granulomatosis and polyangiitis (Churg-Strauss, EGPA): monocentric experiences in 150 patients. Ann Rheum Dis. (2013) 72:1011–7. doi: 10.1136/annrheumdis-2012-201531, PMID: 22887848

[68] Puéchal X Pagnoux C Baron G Lifermann F Geffray L Quémeneur T. Non-severe eosinophilic granulomatosis with polyangiitis: long-term outcomes after remission-induction trial. Rheumatol Oxf Engl. (2019) 58:2107–16. doi: 10.1093/rheumatology/kez139, PMID: 31056661

[69] Terrier B Charles P Aumaître O Belot A Bonnotte B Crabol Y. ANCA-associated vasculitides: recommendations of the French Vasculitis study group on the use of immunosuppressants and biotherapies for remission induction and maintenance. Presse Med. (2020) 49:104031. doi: 10.1016/j.lpm.2020.104031, PMID: 32645418

[70] Maritati F Alberici F Oliva E Urban ML Palmisano A Santarsia F. Methotrexate versus cyclophosphamide for remission maintenance in ANCA-associated vasculitis: a randomised trial. PLoS One. (2017) 12:e0185880. doi: 10.1371/journal.pone.0185880, PMID: 29016646 PMC5634660

[71] Caminati M Maule M Bello F Emmi G. Biologics for eosinophilic granulomatosis with polyangiitis. Curr Opin Allergy Clin Immunol. (2023) 23:36. doi: 10.1097/ACI.0000000000000875, PMID: 36413432

[72] Basta F Mazzuca C Nucera E Schiavino D Afeltra A Antonelli IR. Omalizumab in eosinophilic granulomatosis with polyangiitis: friend or foe? A systematic literature review. Clin Exp Rheumatol. (2020) 38:214–20. 32083537

[73] Celebi Sozener Z Gorgulu B Mungan D Sin BA Misirligil Z Aydin O. Omalizumab in the treatment of eosinophilic granulomatosis with polyangiitis (EGPA): single-center experience in 18 cases. World Allergy Organ J. (2018) 11:39. doi: 10.1186/s40413-018-0217-0, PMID: 30524647 PMC6276141

[74] Solans-Laqué R Fraile G Rodriguez-Carballeira M Caminal L Castillo MJ Martínez-Valle F. Clinical characteristics and outcome of Spanish patients with ANCA-associated vasculitides: impact of the vasculitis type, ANCA specificity, and treatment on mortality and morbidity. Medicine (Baltimore). (2017) 96:e 6083. doi: 10.1097/MD.0000000000006083 PMC5569416 28225490

[75] Keogh KA Specks U. Churg-Strauss syndrome: clinical presentation, antineutrophil cytoplasmic antibodies, and leukotriene receptor antagonists. Am J Med. (2003) 115:284–90. doi: 10.1016/S0002-9343(03)00359-0, PMID: 12967693

[76] Solans R Bosch JA Pérez-Bocanegra C Selva A Huguet P Alijotas J. Churg–Strauss syndrome: outcome and long-term follow-up of 32 patients. Rheumatology. (2001) 40:763–71. doi: 10.1093/rheumatology/40.7.763, PMID: 11477281

[77] Della Rossa A Baldini C Tavoni A Tognetti A Neglia D Sambuceti G. Churg–Strauss syndrome: clinical and serological features of 19 patients from a single Italian Centre. Rheumatology. (2002) 41:1286–94. doi: 10.1093/rheumatology/41.11.1286, PMID: 12422002

[78] Booth AD Almond MK Burns A Ellis P Gaskin G Neild GH. Outcome of ANCA-associated renal vasculitis: a 5-year retrospective study. Am J Kidney Dis. (2003) 41:776–84. doi: 10.1016/S0272-6386(03)00025-8, PMID: 12666064

[79] Bourgarit A Toumelin PL Pagnoux C Cohen P Mahr A Guern VL. Deaths occurring during the first year after treatment onset for polyarteritis nodosa, microscopic polyangiitis, and Churg-Strauss syndrome: a retrospective analysis of causes and factors predictive of mortality based on 595 patients. Medicine (Baltimore). (2005) 84:323. doi: 10.1097/01.md.0000180793.80212.17, PMID: 16148732

